# High VEGF with Rapid Growth and Early Metastasis in a Mouse Osteosarcoma Model

**DOI:** 10.1155/2007/95628

**Published:** 2007-11-26

**Authors:** Shang-You Yang, Haiying Yu, Jeffrey E. krygier, Paul H. Wooley, Michael P. Mott

**Affiliations:** Department of Orthopaedic Surgery, Wayne State University, Detroit, MI 48201, USA

## Abstract

A murine model of osteosarcoma was developed to investigate the association between the expression of VEGF and the progression of osteosarcoma. Two human osteosarcoma cell lines with distinct VEGF expressions were introduced into proximal tibiae of immuno-deficient SCID mice, either by direct injection through the cortical bone or surgical exposing and drilling on the tibial metaphysis to seed tumor cells. Bone tumors were obvious on microCT within 4 weeks following osteosarcoma cell inoculation through surgical delivery. In contrast, direct injection without drilling often resulted in periosteal tumors. Although neoplasms were developed regardless of VEGF levels, orthotopic tumors derived from high VEGF-expressing cells were detected 2 weeks earlier on CT images than the ones from VEGF negative cells. At sacrifice, high VEGF tumors were distinctively larger in size and more frequently invaded the adjacent bone tissue. Multiple metastatic lesions were found in all the lung tissues at 8 weeks from high VEGF group, whereas only 1 of 7 VEGF negative tumors exhibited pulmonary metastasis. Overall, this model developed with the surgical tumor cell delivery results in histological and radiographic features more consistent with primary osteosarcoma. Interestingly, VEGF expression correlates with the early establishment, rapid tumor growth, and the development of pulmonary metastasis.

## 1. INTRODUCTION

Osteosarcoma is a highly malignant bone tumor that usually affects adolescents and young adults. Multimodality treatment consisting of aggressive adjuvant chemotherapy and wide surgical resection has markedly improved prognosis in osteosarcoma. However, nearly a half of the patients still experience therapeutic failures [1]. Treatment of patients with osteosarcoma, especially those nonresponsive to current chemotherapy protocols, remains a major challenge. Although the exact mechanisms of invasion into adjacent tissues, the microcirculatory system, and lymphogenic or hematogenic dissemination with subsequent extravasation and formation of secondary tumor foci remain unclear, it appears that high metastatic potential and recurrence rate are associated with promoted neovascularization [2]. Indeed, growth, invasion, and metastasis of solid tumors are dependent on neovascularization, which is regulated through angiogenesis [3]. The development of tumor angiogenesis is believed to be dependent on the net balance between the actions of angiogenesis promoters and inhibitors. Proangiogenic factors are upregulated in malignancy, and have been linked to poor prognosis with disease progression [4]. It appears that the proangiogenic factors within solid tumors stimulate host vascular endothelial cell mitogenesis and possibly chemotaxis. A large number of proangiogenic factors have been identified, including basic fibroblast growth factor (bFGF), platelet-derived growth factor (PDGF), transforming growth factor beta-1 (TGFβ1), transforming growth factor alpha (TGFα), and epidermal growth factor (EGF) [5]. Perhaps the best characterized one is vascular endothelial growth factor (VEGF), which is relatively unique among growth factors in terms of its specificity for the vascular endothelium [6]. Enhanced VEGF gene expression has been identified in a number of malignant tumors from breast, lung, ovarian, liver, and colon in comparison with normal tissue [7]. However, there are few studies aimed at elucidating the correlation of VEGF expression and progression of osteosarcoma. The objectives of the current study are to characterize a murine model of human osteosarcoma with distinct VEGF expressions to monitor VEGF expression in the development and progression of this most common malignant bone tumor.

## 2. MATERIALS AND METHODS

### 2.1. Animals

Severe combined immunodeficient (SCID) mice at four weeks of age were obtained from the Jackson Laboratory (Bar Harbor, Me, USA) and used as hosts for the experimental sarcoma. The animals were housed in a pathogen-free environment and given free access to autoclaved chow and water. All mice were quarantined for one week prior to experimentation.

### 2.2. Osteosarcoma cell lines

Human osteosarcoma cell lines, G-292 (CRL-1423) and HOS (CRL-1543) cells were obtained from American Type Culture Collection (Manassas, Va, USA) and processed according to the vendor’s instruction. G-292 Cells were cultured in McCoy’s 5a medium with 1.5 mM L-glutamine and 2.2 g/L sodium bicarbonate, while HOS cells were maintained in MEM (Eagle) medium with 2 mM L-glutamine and 1.5 g/L sodium bicarbonate, 0.1 mM nonessential amino acids, and 1.0 mM sodium pyruvate. Both cell cultures also contained 10% fetal bovine serum (FBS), 100 U/mL penicillin, and 100 μg/mL streptomycin. Cells were incubatedat 37C° in a 5% CO_2_ incubator. Media were changed every 3 days. When 90% confluence was reached, culture medium was removed, the cell layer rinsed thoroughly with phosphate buffered saline (PBS), and enzymatically dissociated by adding 0.25% (w/v) trypsin-0.03% (w/v) EDTA. Cells were maintained by subculture at a ratio of 1 : 8. Prior to implantation, the cell suspensions were diluted with 0.5% (w/v) trypan blue in 0.16 mol/L ammonium chloride to assess cell viability and number.

### 2.3. Enzyme linked immunosorbent assay (ELISA)

Human VEGF levels in the culture medium collected from G-292 (CRL-1423) and HOS (CRL-1543) cell cultures were assessed using Quantikine ELISA kits (R&D Systems) with a pair of rabbit anti-human VEGF antibodies (R&D Systems) according to manufacture’s instruction and the standardized protocol previously described [8].

### 2.4. Establishment of the orthotopic osteosarcoma

The Institutional Animal Investigation Committee approved all animal procedures. SCID Mice were anesthetized by IP injection of a mixture of Xylazine (8 mg/kg) and Ketamine (100 mg/kg). Two different approaches were utilized to deliver the osteosarcoma cells to the right tibiae of the mice.

In the surgical exposure group, under strict sterile conditions, a 0.5 cm incision along the lateral collateral ligament of the knee was made to expose the proximal of tibia. A 0.8 mm dental drill was used to drill a small hole across the metaphysis. The wound was rinsed with PBS containing Penicillin G (500 unit/mL) and Streptomycin (500 μg/mL) before closing the skin cut by simple interrupted sutures. 100 μL of culture medium containing 10^6^ osteosarcoma cells (G-292 or HOS) was injected into the hole immediately after surgery. The other limb received a sham operation without injection of tumor cells.

To compare with the surgical groups, mice were given a direct injection of 10^6^ G-292 or HOS cells through the cortical bones into the proximal tibial metaphysis using a 23 G needle, without the surgical procedures described above. All animals were monitored throughout the study with daily visual inspection for general health and tumor development. A caliper was used to measure the leg dimensions to assess growth of the tumor. Animals were sacrificed at 6 and 8 weeks after tumor cells inoculation by CO_2_ asphyxiation. Legs containing orthotopic tumors, liver, and lungs were harvested for histological and molecular evaluations.

### 2.5. MicroCT evaluation

An eXplore Locus MicroCT system (GE Medical Systems, London, ON, Canada) was used to monitor the tumor growth, characters of bone lesions, and lung metastasis. Mice were scanned immediately following tumor cell implantations, and every 2 weeks thereafter. All mice were fully anesthetized (10 mg/kg of Xylazine and 120 mg/kg of Ketamine) and restrained during each CT scanning. Scan parameters were set at 45 μm isotropic voxel size, 400 projections, 400 milliseconds exposure time, 80 KW voltages, and 450 μA current.

### 2.6. Histology process and immunohistological (IHC) examination

Proximal tibiae bearing osteosarcoma tissue and receiving sham operation were collected along with the lungs and liver at sacrifice. Tissues were fixed in buffered formalin, decalcified in EDTA (bone tissue), and embedded in paraffin at consistent orientation. The bone tumors in tibia were cut longitudinally and other tissues were cut in multiple layers. All tissues were stained with hematoxylin and eosin, and examined under a Zeiss light microscope. Digital photomicrographs were captured and analyzed using Image-Pro Plus analysis software (Media Cybernetics, Silver Spring, Md, USA). Tumor length and width were measured, and metastatic lesions were evaluated.

IHC was performed on primary orthotopic tumor sections to detect the expression levels of VEGF, Flt-1 (physiological receptor of VEGF), and CD31, according to the instructions of the vender and the protocol published previously [9]. Briefly, paraffin sections were deparaffinized in xylene and rehydrated in graded alcohols and water. 0.3% Hydrogen Peroxide was applied to diminish endogenous peroxidase followed by microwave incubation to enhance the antigen. After blocking with 1.5% normal goat serum for 1 hour, the sections were incubated overnight with the primary antibodies (2 μg/mL, BD, Pharmingen) in a moisturized chamber at 4C°. Biotin-conjugated secondary antibody and Avidin-biotin enzyme reagents were sequentially applied for 30 minutes between extensive washes. The color was developed by adding $3.3^{\prime }$-diaminobezidine tetrahydrochloride (DAB). In negative control sections, an irrelevant antiserum was applied at the same concentration as the primary antibody. Digital images were captured and analyzed using the Image-Pro software package. The level of positive staining and localization was evaluated in six different fields and expressed as pixel density.

### 2.7. RNA extraction and real-time quantitative PCR for gene expression

Primary osteosarcoma tissues including the adjacent bone were snapped-frozen in liquid nitrogen at the time of sacrifice. The tumor tissues were ground into power while deep-frozen and a portion of the tumor-bone powder homogenized in 0.5 mL of Trizol solution (Gibco BRL) using a glass Grinder Pestle. Total RNA extraction was performed using a commercial kit (Tel-Test Inc., Friendswood, Tex, USA) in accordance with the manufacturer instructions. The precipitated RNA was then treated with DNase and passed through a spin column (Rneasy mini kit, Qiagen) for further purification. Reverse transcription and real-time PCR for the expression of VEGF, *c-myc*, and *c-fos* was performed as described previously [10]. Briefly, cDNA was reverse transcribed from 0.5 μg of total RNA in 40 μL reaction mixture containing 1× PCR buffer, 500 μM each of deoxynucleotide triphosphates (dNTP), 0.5 U/μL of RNase inhibitor, 2.5 μM random hexamers, 5.5 mM MgCl2, and 1.25 U/μL of reverse transcriptase (Perkin Elmer, Conn, USA); and incubated in a DNA Thermal Cycler (Perkin Elmer, Mass, USA) at 25C° for 10 minutes, 48C° for 5 minutes followed by 95C° for 5 minutes. For real-time PCR steps, sense and antisense primer pairs were picked in “Primer3” software (http://frodo.wi.mit.edu/cgi-bin/primer3/primer3_www.cgi) and listed as follows: VEGF $5^{\prime }$-AAGGAGGAGGGCAGAATCAT-$3^{\prime }$ and $5^{\prime }$-ATCTGCATGGTGATGTTGGA-$3^{\prime }$; *c-myc* $5^{\prime }$-GGTGGAAAACCAGGTAAGCA-$3^{\prime }$ and $5^{\prime }$-CCTTCTCCTCTGCCATCTTG-$3^{\prime }$; *c-fos* $5^{\prime }$-AAGGAGAATCCGAAGGGAAA-$3^{\prime }$ and $5^{\prime }$-AGGGCCCTTATGCTCAATCT-$3^{\prime }$. To standardize target gene level with respect to variability in RNA and cDNA quality, house keep gene 18S was coamplified as an internal control. Reaction mixtures of 25 μL containing 12.5 μL of 2× SYBR Green PCR Master Mix (5 mM MgCl_2_, 200 μM dATP, dCTP, dGTP, 400 μM dUTP, 1.25 U AmpliTaq Gold DNA polymerase, 0.5 U AmpErase uracil N-glycosylase), 0.5 μL each of 0.4 μM target primers and 2 μL of cDNA. The PCR reactions were set in MicroAmp optical 96-well reaction plates with MicroAmp optical caps and amplified in the ABI Prism 7700 Sequence Detector (PE-Applied Biosystems, Foster City, Calif, USA) for 40 cycles (95C° for 15 seconds, 60C° for 1 minute). The fluorescent signals were recorded dynamically. The values of threshold cycle (Ct) at which a statistically significant increase in reporter-dye signals (ΔRn) is first detected were imported into Microsoft Excel program and used to calculate the relative quantification of the target gene expression. With the Ct value of 18 S the samples as an internal control and mean Ct value of target gene expression from the bone tissue of the sham operations as the calibrator samples (named 0% expression change), the comparative gene expressions of the experimental tumor groups over the sham controls were calculated according to the formula given in the manufacturer’s manual [11].

### 2.8. Statistical analyses

A total of 40 mice were used for this study with 2 premature deaths (anesthetics overdose, eliminated from the study). Statistical analysis between different VEGF expressing tumor groups was performed by Student t-test, or the ANOVA test; with the Schafer formula for post hoc multiple comparisons, using the SPSS software package (SPSS. Chicago, Ill, USA). A *P* value of less than .05 was considered as significant difference. Data are expressed as mean ± standard error of the mean.

## 3. RESULTS

### 3.1. VEGF expression in osteosarcoma cell lines

 ELISA was performed to determine VEGF expression levels in medium after 72 hours of cell culture. There were only trace levels of VEGF expressed in the culture media collected from the dishes of the HOS (CRL-1543) cells. However, the VEGF level in media of G-292 (CRL-1423) cells was over 27 times higher than the level detected in media of HOS cells during the same culture period of time.

### 3.2. Primary orthotopic osteosarcoma growth

**Figure 1:**
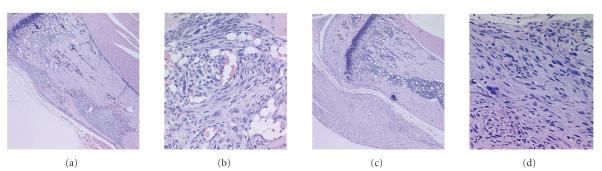
Two methods to establish the orthotopic tumors. Panel (a) shows surgical exposure of the proximal tibia plus drilling a hole through cortical bone to accommodate osteosarcoma cells (25× magnification); Panel (b) shows a high magnification (200×) of orthotopic bone tumor with the surgical method. Panels (c) and (d) example the tumor by direct injection of tumor cells to proximal tibia, mostly out growth surrounding the bone (25× and 200×).

Regardless of the VEGF expression, osteosarcoma cells from both lines induced orthotopic tumors in the upper tibia region of the SCID mice. Daily measurement of the legs for tumor growth in tibia did not generate meaningful data until the late stage of the tumorigenesis, and no differences were observed between the two tumor cells lines (data not shown), possibly due to the incarcerated tumor expansion in bone tissues. On the other hand, the different methods of tumor cell inoculation resulted in varying growth patterns of the orthotopic tumors. Surgical exposure and drilling of the tibia promoted cell anchoring and resulted in intramedullary lesions (Figures 1(a) and 1(b)). Any surrounding soft tissue bulging was not obvious until 6 weeks after cell transplantation. Tumors established using direct injection of osteosarcoma cells resulted in primary tumors anchored to adjacent periostea and grown away from the bone surface (Figures 1(c) and 1(d)).

### 3.3. Rapid growth of the tumors derived from high VEGF cells

**Figure 2:**
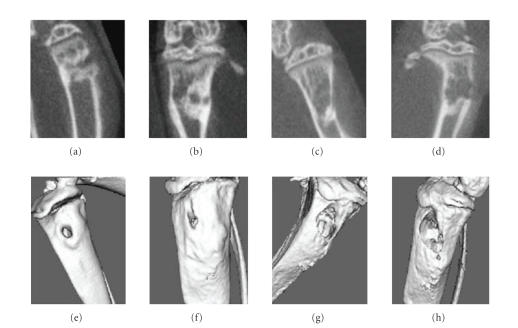
MicroCT images of the orthotopic osteosarcoma model. Panels (a) and (e) shows 7 days after G292 tumor cells inoculation; (b) and (f) show 2 weeks; Panels (c) and (g) show 4 weeks; and panels (d) and (h) show 8 weeks after the tumor cells transfer. Lower panels show the 3D isosurfaces of the tibia harboring tumors.

 MicroCT indicated that 6 of 7 mice surgically implanted with G292 cells (high VEGF expression) developed detectable bone tumors at 2 weeks after tumor cell inoculation, whereas the HOS cells-(low VEGF expression) derived tumors became detectable in microCT images at either 4 weeks or 6 weeks after inoculation (P<.05). Periodical microCT scans dynamically monitored the development and growth of the primary tumor and proved a useful assessment technique in this study. Figure 2 reveals an example of the progression of the experimental osteosarcoma (G292 cells).

**Figure 3:**
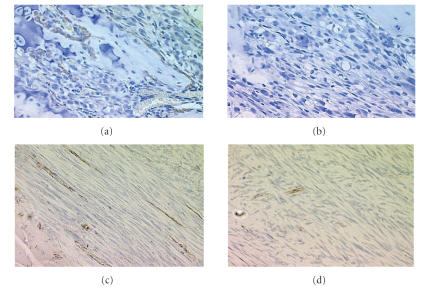
Immunohistochemical staining of the orthotopic osteosarcoma models. Micrographs (a) and (c) were tumors derived from high VEGF G-292 cells; while (b) and (d) were sample from HOS cell transplantation. (a) and (b) were stained with anti-human VEGF antibodies; and (c) and (d) were against anti-human CD31.

Histology evaluation using a computerized image analysis system showed that the average sizes (observed areas on sections) of the primary tumors derived from G292 cells were 3.97±0.84×2.95±0.24 mm (length × width), significantly larger than HOS experimental orthotopic tumors (1.85×1.34) mm at 8 weeks, the termination point of the experiment (P<.05). In addition, immunohistochemical staining revealed strong positive CD31 (Figure 3(a)) and VEGF (Figure 3(c)) staining on G292 cell-derived orthotopic tumor sections, in comparison with the tumors from HOS cells (Figures 3(b) and 3(d)).

### 3.4. Molecular assessment

**Figure 4:**
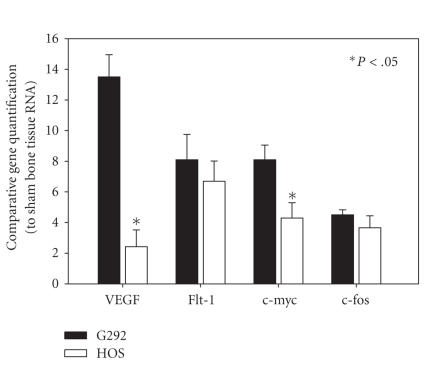
Comparative gene expression quantification using sham operated bone tissue as controls. Data expressed as the relative expressions over those from sham bone tissue (*P<.05).

 RNA samples isolated from the orthotopic tumors were examined for the gene expression of VEGF, Flt-1, and both oncogenes *c-fos* and *c-myc.* Relative gene expressions over the sham controls were calculated. Significant higher VEGF expression was companioned with elevated *c-myc* expression in G292-derived primary tumors (Figure 4).

### 3.5. Remote tumor metastasis

**Figure 5:**
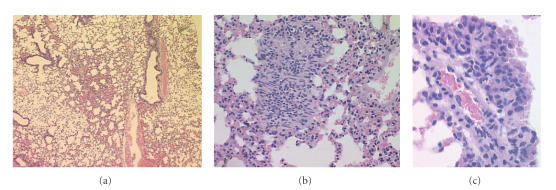
Pulmonary metastasis of the experimental osteosarcoma established by G292 cells. Lung sections stained with H & E. Panel (a) is a micrograph at 25× magnification, (b) shows the metastatic lesion at 200×, while panel (c) at 400× magnification.

Lungs and liver were collected and pathologically examined for tumor metastasis. No emerging metastatic lesions were identified in liver tissues. However, all of the G292-experimental osteosarcoma mice sacrificed at 8 weeks and 80% of the mice at 6 weeks developed extensive lung metastasis lesions (Figure 5). In comparison, only 1 out of 7 HOS-tumor mice developed a similar pulmonary change within the same time.

## 4. DISCUSSION

The current study establishes a mouse model of osteosarcoma by introducing human osteosarcoma cells to the common disease site-proximal tibia-in immune deficiency mice. Using two clonally unrelated human osteosarcoma cell lines, we try to compares the correlation between VEGF expression and the development and progression of osteosarcoma in this murine model. Osteosarcoma is an extremely aggressive malignant tumor of the skeleton characterized by fast growth and early hematogenic metastasis. It is postulated that the growth, invasion, and metastatic potential of many solid tumors are dependent on angiogenesis mediated via the potent proangiogenic factor VEGF. Clinical studies have shown that the density of intratumoral microvessels correlates well with the grade of invasiveness, the frequency of metastasis, and clinical prognosis in many types of cancers [12, 13]. Malignancies have an absolute requirement for a persistent supply of new blood vessels to nourish their growth and to facilitate metastasis. The angiogenic cascade leading to tumor vascularization can be divided into two general phases, the prevascular phase and the vascular phase [5, 14]. Once tumor cells transform into angiogenic phenotype, these malignant cells in avascular tumors become capable of inducing neovascularization, which permits a rapid rate of tumor growth and increases metastatic potential. The development of tumor angiogenesis is believed to be dependent on the elevated expression and presence of active proangiogenic factors within the solid tumors that stimulate host vascular endothelial cell mitogenesis and possibly chemotaxis [4]. VEGF (the most potent direct-acting proangiogenic protein known) is a diffusible endothelial cell-specific mitogen and an angiogenic factor that also increases vascular permeability. It stimulates and maintains neovascularization in a variety of tumor types [6]. In situ hybridization assays have shown a marked upregulation of VEGF mRNA in many human tumors [15], and VEGF mRNA has been found to be much more abundant in cancer cells than in endothelium, suggesting that the cancer cells themselves generate VEGF to induce angiogenesis through a paracrine loop. However, little is known about the role of angiogenesis and proangiogenic factors, such as VEGF, in the development and biologic activity of malignant bone tumors [16] though several clinical studies have found that many osteosarcoma patients with pulmonary metastasis had primary tumors with high levels of VEGF expression [17]. In this study, we utilized established osteosarcoma cell lines with distinct VEGF expressions to generate an experimental orthotopic osteosarcoma mouse model. The data clearly show that the orthotopic tumors derived from high VEGF expressing osteosarcoma cells (G292) grew more rapidly and were more likely to metastasize to the lung by 6–8 weeks. In contrary, the experimental orthotopic tumors derived from low VEGF expressing cells were less inclined toward early pulmonary metastasis. The data provides a VEGF targeting osteosarcoma model to all further studies to understand the role of VEGF in the pathogenesis, progress, and prognosis of the tumor.

With the recent expansion of molecular biology techniques, genetic alterations, associated the development and metastases of malignant tumors, have been observed. Cellular oncogenes have been found to be activated by DNA rearrangements (proviral insertions, chromosome translocations, and DNA amplifications). These alterations may result in an increased or deregulated gene expression. Although numerous oncogenes and tumor suppressor genes have been identified in osteogenic sarcomas, *c-myc* and *c-fos* seem to be expressed with a relatively high incidence [18–22]. *C-myc* protooncogene on chromosome 8 encodes transcription factor, which involves in the regulation of cell growth, DNA replication, and transcriptional regulation of specific target genes [23]. *C-fos* protooncogene, the cellular homologue of *v-fos*, is involved in osteoblast and chondrocytes differentiation [21]. Studies have indicated that *c-myc* and *c-fos* were found overexpressed in the relapse osteosarcoma and the metastasis cases [19]. In this G292 experimental osteosarcoma model, these oncogenes, especially *c-myc*, markedly expressed in the tumor tissues supplementing with the high VEGF expression.

To our knowledge, there are only a few reports concerning experimental murine models of osteosarcoma [24, 25]. Luu et al. recently reported an orthotopic osteosarcoma model by injecting three related human osteosarcoma lines (TE85, MNNG/HOS, and 143 B) to proximal tibia of athymic nude mice [25]. Coincidentally, the authors used the N-methyl-N′-nitro-N-nitrosoguanidine (MNNG) transformed HOS osteosarcoma cells in their model and found that the cells resulted in tumorigenesis in primary injection site, but significant less pulmonary metastasis compared to the other cell line (143 B). Since MNNG is known to create DNA strand breakage, it is postulated that the mutagen may cause the genetic alteration and be responsible for the phenotypic difference in tumor growth and metastatic potential [25]. We utilized the original HOS cells in our model, and predictable primary tumors were established in upper tibiae of SCID mice. However, the pulmonary metastasis was not evident, in agreement with Luu’s findings [25]. One of the major characteristics of HOS cells is their low expression of VEGF, that is, experimental evaluations have been performing to test the hypothesis that the slower neo-angiogenesis in primary tumors due to lack of VEGF contributes to the delayed remote metastasis of osteosarcoma.

Kaya et al. [17] examined 27 human cases of primary osteosarcomas and evaluated the correlation between the expression of VEGF and presence of microvessels, the clinical-pathological variables, and the survival of patients. Their investigation suggested that patients with VEGF-positive osteosarcoma developed a significantly higher lung metastasis rate and therefore a reduced chance of survival. It appears that antiangiogenic therapy, especially the blockade of VEGF effects may be a novel and potentially promising strategy for this common form of malignant tumor in orthopaedic surgery. The VEGF-associated osteosarcoma mouse model we are reporting here may provide a platform to screen potential interventions, including antiangiogenic gene therapy, for osteosarcoma.

## References

[1] Rosen G, Suwansirikul S, Kwon C (1974). High dose methotrexate with citrovorum factor rescue and adriamycin in childhood osteogenic sarcoma. *Cancer*.

[2] Coomber BL, Denton J, Sylvestre A, Kruth S (1998). Blood vessel density in canine osteosarcoma. *Canadian Journal of Veterinary Research*.

[3] Folkman J (1995). Angiogenesis in cancer, vascular, rheumatoid and other disease. *Nature Medicine*.

[4] Folkman J (2002). Role of angiogenesis in tumor growth and metastasis. *Seminars in Oncology*.

[5] McMahon G (2000). VEGF receptor signaling in tumor angiogenesis. *The Oncologist*.

[6] Leung DW, Cachianes G, Kuang W-J, Goeddel DV, Ferrara N (1989). Vascular endothelial growth factor is a secreted angiogenic mitogen. *Science*.

[7] Lee YH, Tokunaga T, Oshika Y (1999). Cell-retained isoforms of vascular endothelial growth factor (VEGF) are correlated with poor prognosis in osteosarcoma. *European Journal of Cancer*.

[8] Yang S-Y, Wu B, Mayton L, Evans CH, Robbins PD, Wooley PH (2002). IL-1Ra and vIL-10 gene transfer using retroviral vectors ameliorates particle-associated inflammation in the murine air pouch model. *Inflammation Research*.

[9] Ben-Josef E, Yang S-Y, Ji TH (1999). Hormone-refractory prostate cancer cells express functional follicle-stimulating hormone receptor (FSHR). *The Journal of Urology*.

[10] Yang S-Y, Wu B, Mayton L (2004). Protective effects of IL-1Ra or vIL-10 gene transfer on a murine model of wear debris-induced osteolysis. *Gene Therapy*.

[11] PE Applied Biosystems (1998). TaqMan cytokine gene expression plate I—protocol.

[12] Weidner N, Carroll PR, Flax J, Blumenfeld W, Folkman J (1993). Tumor angiogenesis correlates with metastasis in invasive prostate carcinoma. *American Journal of Pathology*.

[13] Weidner N, Semple JP, Welch WR, Folkman J (1991). Tumor angiogenesis and metastasis—correlation in invasive breast carcinoma. *The New England Journal of Medicine*.

[14] Hanahan D, Folkman J (1996). Patterns and emerging mechanisms of the angiogenic switch during tumorigenesis. *Cell*.

[15] Suzuki K, Hayashi N, Miyamoto Y (1996). Expression of vascular permeability factor/vascular endothelial growth factor in human hepatocellular carcinoma. *Cancer Research*.

[16] Holzer G, Obermair A, Koschat M, Preyer O, Kotz R, Trieb K (2001). Concentration of vascular endothelial growth factor (VEGF) in the serum of patients with malignant bone tumors. *Medical and Pediatric Oncology*.

[17] Kaya M, Wada T, Akatsuka T (2000). Vascular endothelial growth factor expression in untreated osteosarcoma is predictive of pulmonary metastasis and poor prognosis. *Clinical Cancer Research*.

[18] Barrios C, Castresana JS, Ruiz J, Kreicbergs A (1993). Amplification of *c*-*myc* oncogene and absence of *c*-*Ha*-*ras* point mutation in human bone sarcoma. *Journal of Orthopaedic Research*.

[19] Gamberi G, Benassi MS, Bohling T (1998). *C*-*myc* and *c*-*fos* in human osteosarcoma: prognostic value of mRNA and protein expression. *Oncology*.

[20] Pompetti F, Rizzo P, Simon RM (1996). Oncogene alterations in primary, recurrent, and metastatic human bone tumors. *Journal of Cellular Biochemistry*.

[21] van den Berg S, Rahmsdorf HJ, Herrlich P, Kaina B (1993). Overexpression of *c*-*fos* increases recombination frequency in human osteosarcoma cells. *Carcinogenesis*.

[22] Wu J-X, Carpenter PM, Gresens C (1990). The proto-oncogene *c*-*fos* is over-expressed in the majority of human osteosarcomas. *Oncogene*.

[23] Cole MD, McMahon SB (1999). The Myc oncoprotein: a critical evaluation of transactivation and target gene regulation. *Oncogene*.

[24] Khanna C, Prehn J, Yeung C, Caylor J, Tsokos M, Helman L (2000). An orthotopic model of murine osteosarcoma with clonally related variants differing in pulmonary metastatic potential. *Clinical and Experimental Metastasis*.

[25] Luu HH, Kang Q, Park JK (2005). An orthotopic model of human osteosarcoma growth and spontaneous pulmonary metastasis. *Clinical and Experimental Metastasis*.

